# Prosthetic Dentistry

**Published:** 1884-01

**Authors:** C. B. Porter


### COMMUNICATIONS.

### Prosthetic Dentistry.

BY C. B. PORTER.

Read before the Michigan State Dental Society.

MR. PRESIDENT AND GENTLEMEN: I am glad and thank the Executive Committee for again bringing forward the subject of Prosthetic Dentistry.

If we wish to encourage this important branch of dentistry, and desire to see it brought to that standard of excellence, beauty, and usefulness that it deserves, we *must* give it a proper place in our dental discussions. That there is room for improvement, I think none will deny.

It is unnecessary for me to speak of the preliminary work of Mechanical Dentistry, such as taking impressions, making swages, or the relative value of base plates. But let us consider the human face, its needs when misfortune overtakes it, by the loss of the natural teeth.

Those who have made themselves acquainted with the anatomy of the face, and the physiognomy and temperaments of individuals and peoples, have laid a good foundation upon which to build.

In order to proceed correctly we must have a *definite* plan. We may observe passing faces forever without profit, unless we reduce our observations to practice.

Taking a front view of the head and face, we see marked differences in the form of the outline thus presented by different individuals. The variety is very great, no two being exactly alike; yet we need not be amazed at this almost infinite variety, for, by a careful and close study of faces, we find they are readily and naturally reducible to three classes—namely:

1. The oblong faces.
2. The round faces.
3. The pyriform faces.

There are, of course, many modifications of these three forms of faces, resulting from the different proportions of the tempermental elements combined in each individual; and we may also include age, health, and other physiological conditions and circumstances.

In serving the edentulous we must take all these things into account if we intend to lay any claim to art and science in prosthetic dentistry. The ancient artists found it necessary to adopt a standard of human facial beauty. We see it in all the old Egyptian works of art; also in the Grecian where they have preserved their ideas wrought in the hard rock.

Although three thousand years or more have passed since a standard of proporti ns for correct painting or sculpturing the human face was adopted, artists, from time to time, have endeavored to modify or correct it, still it exists the same, and I hope to show that this idea of a standard of a typical face is no whim but an absolute necessity in prosthetic dentistry.

Let us imagine, if you please, a patient before us—a well-formed man—who has lost none of what we may call personal beauty from the root of nose upward to the crown of the head. Perhaps age shows the marks of many winters upon him, but there is a beauty in even old age; but from the tip of the nose to the point of the chin, a great change has taken place, caused by the entire loss of the teeth, both upper and lower, the alveolar ridge much absorbed, sunken lips and cheeks.

Now let us consider what course we had best pursue to serve our patient best. The preliminary steps are about the same in all cases. Should we not, just here, study his habits, his modes of thought and expression, and if our patient happens to be of finer mould than common humanity, the time now consumed is not lost if rightly directed. Measure the face by a true standard and note its loss. But what is a *true* standard, a measured outline of facial equality?

If we draw a straight vertical line and cut it into four equal parts, the horizontal line marked 1 shows the top of the head; 5, the point of the chin; 2, the root of the hair; 3, the root of the nose; 4, the point of the nose. The lower one-quarter must be divided into four equal parts. The first $\frac{1}{4}$ indicates position of the mouth; the second $\frac{1}{4}$, length of lower lip, and the last $\frac{1}{2}$ the length of chin.

In attempting to commit my thoughts to paper, I find my pen far too slow to keep pace with my ideas, or correctly record the spark of enthusiasm I feel on this subject, consequently if I come before you with a disjointed, unfinished paper when the subject is so large, I trust I will have your indulgence and pardon.

But to return to the subject from which I have wandered. Now we have our patient, who is edentulous, before us. If we would be successful, we must make a careful note of the irregularities of the facial circle.

Of the three types he will nearest resemble one of them, it may be the oblong, possibly the pyriform type whatever it may be. For, any piece of work to be successful in the restoration of the contour of the face, it will be necessary that it shall be in harmony with the features. Think back how our patient looked many years ago; restore him in our mind's eye, just as an architect would an old ruin, as he takes the measurement of what there is left of its former magnificence and grandeur, and reconstructs it by drawings until you see it in its former beauty; so with our patient. Take him as he is, and build up and restore by modeling your wax as the artist does clay, till his old schoolmates, who have not met him for many years, will look upon him as one remarkably well kept, and on whom his years rest lightly. To attain this we must observe the general conformation of the face; make this and our work agree. To do this we must exercise care and judgment.

Artificial teeth, as they come from the manufacturers, should represent age and temperaments—perhaps nationality—as natural teeth do, and of tints and sizes, a very large variety to suit all manner and conditions of men and women.

We have the patient and dental materials in all their numerous forms and colors before us. We must now bring into use all the skill and judgment we possess in selecting colors, sizes, and form of teeth, that best correspond with age, color of skin, eyes, and hair; in other words, the general make-up and temperament of our patient. When we have so far succeeded—possibly to our satisfaction—we must not relax our efforts to harmonize the whole, or we will ignominously fail. Much thought and labor must now be bestowed to get a proper adjustment of upper and lower dentures. This may be done in an early stage of the work by modeling with wax; still I like to have my patient near at this stage to make a careful comparison, to see, with the dentures *in situ*, if I have made a proper estimate of the loss of structure by absorption that my work shall only supply the loss. Just here I would warn the young in the profession to be careful and not cultivate false ideas, for they are hard to eradicate.

The dentist, in his daily dealings with individuals, has a fine opportunity to study people of various races and nationalities, enabling us to classify the different forms of head, brain, and face. But the more we study the face, the more it becomes apparent to us that the *mouth* is the most eloquent feature of it—the center of expression.

I cannot better describe what true dental art is, and how it should harmonize with the individual for whom the work is made, than by relating a case in actual life.

Dr. A—— was employed by Mrs. B—— to make for her a full set of teeth. The lady was possessed of much natural beauty. To say she was really queen-like in form, magnificent in her very motions and carriage, one who moved in the best society, and was courted for her personal charms and character, is not saying too much. After the teeth were completed, and the lady had attained complete mastery of them, she (now being a widow) wished to visit the country of her early life, her sisters, and old school friends. Of course preparations were made for her reception and entertainment, a supposed invalid. But instead of an old broken down woman, you can imagine their surprise, for our widow was tall and beautiful for her age—being a little past the prime of life—her face, of course, older than when she married a young and promising lawyer; her eyes as bright, and her voice as clear as any could wish at fifty; her artificial teeth all that art could accomplish to conceal the artist's work, and render as perfect and natural facial expression as nature herself.

The next case, that of Esq. C——, a man of over fifty, much broken down by hard work, a farmer by occupation. When the dentist first saw him his general outline of face appeared care-worn; cheeks much fallen in; lips flabby; voice very weak; only one tooth remaining, that a lower bicuspid; for twenty years that tooth had stood alone doing service as a good soldier. This, of course, was removed and new teeth inserted. Care was taken to do all that could be done for the gentleman. He was in the habit of buying the best implements for his farm, building the best houses for his grain, and his cattle and horses were sleek and fat, and, as for himself, he wanted artificial teeth for use and personal comfort.

In due time the work was completed, and he set about the task of learning to use them, which it is needless to say he accomplished in due time; and, so successfully was the work done, that none not acquainted with the 'Squire had the most remote idea that he wore artificial teeth. His sunken cheeks and flabby lips were filled out; his voice became stronger; even the dial of his life seemed set back fully fifteen years, and his old friends thought him a hale and a well-kept man on whom advancing age sat lightly.

Let me observe here that one of these was a gold plate, and the other continuous gum. Thousands of just such cases come and go every year.

If we will be successful we must, first of all, give the necessary time to thorough study for such accomplishments; a full and perfect idea of the human face in its normal condition; learn the shape of the bones; study them in detail; learn their processes, ridges, and depressions; also the fullness of the muscles, or their depression; note the shortening of the face from the lower horizontal line; also the depression of the nose. Be careful and avoid an ideal beauty that all faces must conform to.

Just here let me warn those who may be inclined to study the esthetics in dentistry, not to make a rut and fall into it, having succeeded, perhaps, in a limited way, and going no further. In such cases a general sameness will pervade all your work, as it is said of some painters of faces, the same eye may be seen in all their pictures. I would advise to give hours with your patients; laugh with them; talk sense or nonsense; tell stories; any way to beguile them to feel and act natural, that you may bring out and become acquainted with all their habits of facial expression, then your trained mind and hands can do the work so well that every smile and expression shall be as full of meaning as nature herself. Then dental art will have attained its highest glory and usefulness.

